# Association of the polymorphism in NUCB2 gene and the risk of type 2 diabetes

**DOI:** 10.1186/s13098-017-0235-z

**Published:** 2017-05-19

**Authors:** Chunyu Wang, Yulun Wang, Wenchao Hu

**Affiliations:** 1 Department of Clinical Laboratory, Shanxi Provincial People’s Hospital, Taiyuan, China; 2 Department of Endocrinology, Tianjin People’s Hospital, Tianjin, China; 3 Department of Endocrinology, Qilu Hospital of Shandong University (Qingdao), 758 Hefei Road, Shibei District, Qingdao, 266035 Shandong China

**Keywords:** Polymorphism, Nucleobindin 2, Nesfatin-1, Type 2 diabetes

## Abstract

**Background:**

Nesfatin-1, originating from its precursor protein called nucleobindin 2 (NUCB2), plays an important role in glucose metabolism and diabetes. The aim of this study is to examine the association of the c.1012C>G (rs757081) polymorphism of NUCB2 gene with the presence of T2DM.

**Methods:**

This study was performed in a population of 396 patients with T2DM and 196 healthy subjects. The c.1012C>G polymorphism of NUCB2 gene was determined using polymerase chain reaction and sequencing method.

**Results:**

T2DM patients showed lower CG and GG genotype, as well as G allele frequencies compared with healthy subjects. Logistic regression analysis showed that c.1012C>G polymorphism was associated with a decreased risk of developing T2DM. In addition, GG genotype of NUCB2 was significantly correlated with lower levels of body mass index and fasting plasma glucose in patients with T2DM.

**Conclusions:**

The c.1012C>G polymorphism of NUCB2 is associated with the decreased risk of developing T2DM in Chinese Han population.

## Background

Type 2 diabetes mellitus (T2DM) is a chronic metabolic disease characterized by hyperglycemia. In the last few decades, the prevalence of T2DM is increasing rapidly in the world [1]. T2DM causes significant morbidity, disability, and early mortality, and imposes a huge economic burden on the individual, national healthcare, and economy [2]. Both genetic and environmental factors contribute to the incidence of T2DM. T2DM is considered to be a complex multi-gene disorder. A number of genes and their polymorphisms have been shown to contribute to the pathogenesis of this disease [3].

Nucleobindin 2 (NUCB2), containing 396 amino acid residues, is a precursor of nesfatin-1 (residues 1–82), nesfatin-2 (residues 85–163), and nesfatin-3 (residues 166–396) [3]. Nesfatin-1 is a protein comprising 82 amino acids and is highly conserved in humans, rats, and mice [3]. Nesfatin-1 regulates the glucose and energy metabolism in multiple processes. Nesfatin-1 stimulates insulin secretion from pancreatic β-cells, improves insulin sensitivity, and contributes to energy storage [4]. Furthermore, plasma nesfatin-1 concentrations are significantly decreased in T2DM patients compared with healthy controls [5, 6]. These findings suggest the possible role of nesfatin-1 in the pathogenesis of T2DM.

A recent study performed in a population of 1049 obese Caucasian subjects indicated an association of three single nucleotide polymorphisms (SNPs) (rs1330, rs214101 and rs757081) in the NUCB2 gene with obesity [7]. Chen et al. then reported that one SNP of NUCB2 gene, c.1012C>G (Q338E or rs757081) was correlated with childhood adiposity [8]. These data indicate that polymorphisms in the NUCB2 gene could play an important role in the protection against the development of obesity. Obesity is closely correlated with the risk of T2DM. Therefore, it is hypothesized that c.1012C>G polymorphism of NUCB2 may be associated with the risk of T2DM.

The present study is performed to examine the association of c.1012C>G polymorphism of NUCB2 gene with the risk of T2DM in Chinese Han population.

## Methods

### Subjects

This study consisted of 396 patients with T2DM. These patients were diagnosed with T2DM according to the criteria using a fasting plasma glucose (FPG) level ≥7.0 mmol/L or 2-h postprandial plasma glucose (P_2h_PG) level ≥11.1 mmol/L. Patients who had a clinical history of T2DM and were receiving oral hypoglycemic or parental insulin medications were also eligible for this study. However, those with type 1 diabetes were excluded from this study. The control group consisted of 196 normal subjects who had no history of diabetes. None was receiving medication or dietary supplements. All T2DM patients and controls were Chinese Han population.

The study was approved by the Hospital ethics board and all patients provided written informed consent.

### Measurements

Weight, height, systolic blood pressure (SBP), and diastolic blood pressure (DBP) were measured. Venous blood was collected after a minimum of 10 h of fasting. FPG, serum triglycerides (TG), total cholesterol (TC), high-density lipoprotein cholesterol (HDL-C), and low-density lipoprotein cholesterol (LDL-C) were tested using an auto biochemistry instrument (Hitachi 7170, Tokyo, Japan). Body mass index (BMI) was calculated as weight in kilograms divided by height squared in meters (kg/m^2^).

### DNA genotyping

Blood samples were collected from all subjects. Genomic DNA was extracted from peripheral blood using a DNA extraction kit (Qiagen, Valencia, CA). c.1012C>G polymorphism of NUCB2 gene was detected using polymerase chain reaction (PCR) and sequencing method. The primer sequences for genotyping the c.1012C>G polymorphism of NUCB2 gene were as follows: 5′-ATCCTAATGACTTTGACCCC-3′ (forward) and 5′-TGAGGAGACATCTTGCACCAC-3′ (reverse). The PCR was performed with 5 min of a initial denaturation at 95 °C, followed by 35 cycles of 30 s at 95 °C, 45 s at 60 °C and 45 s at 72 °C. A final extension of 72 °C for 7 min completed the reaction. PCR products were directly sequenced by Sangon Biotech Company (Shanghai, China).

### Statistical analyses

Data are presented as mean ± standard deviation (SD). A Chi square test was performed to assess Hardy–Weinberg equilibrium. The value differences between T2DM and control group were compared by Student t test or Chi squared test. Logistic regression analysis was used to determine the risk factors of developing T2DM. The variables of different NUCB2 genotypes among T2DM patients were analyzed using the one-way ANOVA or Chi squared test. A *P* value less than 0.05 was considered statistically significant.

## Results

### Baseline clinical characteristics

As shown in Table 1, T2DM patients showed higher SBP, DBP, FPG, TG, and LDL-C, as well as lower HDL-C than did control subjects.

**Table 1:** Comparison of clinical and laboratory parameters between T2DM patients and control subjects

Characteristics	T2DM group (n = 396)	Control group (n = 196)	*P*
Age (years)	56.57 ± 11.81	56.06 ± 8.96	0.594
Gender (M/F)	210/186	106/90	0.809
BMI (kg/m^2^)	24.96 ± 3.67	24.59 ± 2.91	0.218
SBP (mmHg)	139.18 ± 22.47	121.67 ± 10.77	*<0.001*
DBP (mmHg)	84.17 ± 12.99	77.79 ± 7.38	*<0.001*
FPG (mmol/L)	7.73 ± 1.71	5.00 ± 0.49	*<0.001*
TG (mmol/L)	1.87 ± 1.40	1.54 ± 1.20	*0.004*
TC (mmol/L)	5.20 ± 1.14	5.05 ± 0.84	0.107
HDL (mmol/L)	1.27 ± 0.32	1.45 ± 0.25	*<0.001*
LDL (mmol/L)	3.39 ± 0.96	3.18 ± 0.60	*0.006*

Italic values indicate significance of *P* value (*P* < 0.05)

### Association of c.1012C>G polymorphism with the presence of T2DM

Genotypes of c.1012C>G polymorphism in T2DM patients and control subjects are shown in Table 2. The genotype frequencies of c.1012C>G polymorphism among the controls were in agreement with Hardy–Weinberg equilibrium (*P* = 0.597). The frequency of CG and GG genotypes, as well as G allele was significantly decreased in T2DM patients compared with those in healthy controls (*P* = 0.019 and *P* = 0.004).

**Table 2:** Genotype and allele frequencies of the c.1012C>G polymorphism of NUCB2 gene in T2DM subjects and control subjects

Genotype	T2DM group (n = 396)		Control group (n = 196)		*P* value
	n	%	n	%	
CC	169	42.7	63	32.1	*0.019*
CG	173	43.7	93	47.4	
GG	54	13.6	40	20.4	
C	511	64.1	219	54.5	*0.004*
G	281	35.9	173	45.5	

Italic values indicate significance of *P* value (*P* < 0.05)

### The association of c.1012C>G polymorphism with T2DM

As shown in Table 3, logistic regression analysis showed that c.1012C>G polymorphism was associated with a decreased risk of developing T2DM (OR 0.707, 95% CI 0.554–0.901; *P* = 0.005). After adjusting for age, gender, and BMI, c.1012C>G polymorphism was associated with a decreased risk of developing T2DM (OR 0.704, 95% CI 0.551–0.899; *P* = 0.005). Then a significant association of c.1012C>G polymorphism with T2DM risk was found after adjusting for age, gender, BMI, and serum lipid (OR 0.768, 95% CI 0.589–0.989; *P* = 0.042). However, when adjusted for additional blood pressure, c.1012C>G polymorphism was not associated with the risk of developing T2DM.

**Table 3:** Logistic regression analysis for determining the risk factor of developing T2DM

Characteristics	Model 1		Model 2		Model 3	
	OR (95% CI)	*P*	OR (95% CI)	*P*	OR (95% CI)	*P*
Age (years)	1.002 (0.987–1.018)	0.771	1.011 (0.993–1.029)	0.226	0.990 (0.970–1.009)	0.303
Gender (M/F)	1.102 (0.770–1.577)	0.594	1.555 (1.043–2.320)	*0.030*	1.714 (1.105–2.658)	*0.016*
BMI (kg/m^2^)	1.039 (0.985–1.095)	0.157	0.997 (0.942–1.056)	0.926	0.979 (0.919–1.042)	0.506
SBP (mmHg)	–	–	–	–	1.066 (1.047–1.086)	*<0.001*
DBP (mmHg)	–	–	–	–	0.978 (0.951–1.006)	0.126
TG (mmol/L)	–	–	0.941 (0.763–1.162)	0.573	0.957 (0.772–1.186)	0.690
TC (mmol/L)	–	–	3.247 (1.310–8.050)	*0.011*	2.943 (1.235–7.012)	*0.015*
HDL (mmol/L)	–	–	0.025 (0.008–0.078)	*<0.001*	0.032 (0.010–0.099)	*<0.001*
LDL (mmol/L)	–	–	0.534 (0.211–1.351)	0.185	0.534 (0.222–1.286)	0.162
c.1012C>G polymorphism	0.704 (0.551–0.899)	*0.005*	0.768 (0.589–0.989)	*0.042*	0.773 (0.578–1.032)	0.081

Italic values indicate significance of *P* value (*P* < 0.05)

Model 1: adjust for age, gender, and BMI

Model 2: adjust for age, gender, BMI, and lipid

Model 3: adjust for age, gender, BMI, lipid, and blood pressure

### T2DM patients with different genotypes

The characteristics of different genotypes of c.1012C>G polymorphism in T2DM patients are presented in Table 4. GG genotype was significantly associated with decreased levels of BMI and FPG in T2DM patients. No significant differences in the other characteristics were observed between different genotypes.

**Table 4:** Comparison of characteristics of different genotypes of c.1012C>G polymorphism of NUCB2 gene in T2DM subjects

Characteristics	CC (n = 169)	CG (n = 173)	GG (n = 54)	*P*
Age (years)	57.01 ± 10.69	56.18 ± 12.81	56.41 ± 12.02	0.810
Gender (M/F)	92/77	88/85	30/24	0.742
BMI (kg/m^2^)	25.31 ± 3.76	24.97 ± 3.68	23.81 ± 3.16^a, b^	*0.032*
SBP (mmHg)	138.61 ± 21.99	139.10 ± 22.55	141.20 ± 23.95	0.761
DBP (mmHg)	83.43 ± 14.05	84.51 ± 12.27	85.37 ± 11.85	0.570
FPG (mmol/L)	7.88 ± 1.67	7.78 ± 1.81	7.12 ± 1.36^a, b^	*0.016*
TG (mmol/L)	1.91 ± 1.40	1.81 ± 1.43	1.96 ± 1.37	0.730
TC (mmol/L)	5.24 ± 1.22	5.16 ± 1.14	5.18 ± 0.88	0.792
HDL (mmol/L)	1.25 ± 0.30	1.30 ± 0.35	1.25 ± 0.29	0.348
LDL (mmol/L)	3.41 ± 0.98	3.37 ± 1.01	3.37 ± 0.73	0.915

Italic values indicate significance of *P* value (*P* < 0.05)

^a^Significant versus MetS patients with CC genotype

^b^Significant versus MetS patients with CG genotype

## Discussion

Nesfatin-1/NUCB2 is ubiquitously expressed in peripheral tissues including white adipose tissue. Furthermore, nesfatin-1/NUCB2 is selectively expressed in human and rodent beta cells but absent in alpha, delta, and PP cells [9]. Nesfatin-1/NUCB2 plays a key role in the regulation of glucose metabolism. Nesfatin-1 stimulated glucose-induced insulin secretion from mouse islets and MIN6 cells in a dose-dependent manner [10]. In addition, MIN6 cells showed a four-fold increase of nesfatin-1 release when following the incubation in high glucose compared to low glucose [10]. Riva et al. reported that nesfatin-1 enhanced glucagon secretion but had no effect on insulin secretion from mouse islets or INS-1 cells [9]. On the other hand, nesfatin-1 caused a small increase in insulin secretion and reduced glucose during intravenous glucose tolerance test in mice [9]. And human islet NUCB2 mRNA was up-regulated after cultured in glucolipotoxic conditions [9]. Another study demonstrated that hypothalamic nesfatin-1 infusion could contribute to increased peripheral and hepatic insulin sensitivity by decreasing gluconeogenesis and promoting peripheral glucose uptake in vivo [11]. In addition, inhibition of central nesfatin-1/NUCB2 activity markedly increased food intake and hepatic glucose flux, impaired hepatic insulin sensitivity, and decreased glucose uptake in peripheral tissue in rats [12]. Our study indicated that the GG genotype of c.1012C>G polymorphism was negatively correlated with lower FPG. These results assume an involvement of nesfatin-1 in the development of T2DM.

NUCB2 expression or nesfatin-1 levels were changed during diabetic condition. Both NUCB2 mRNA expression and nesfatin-1 immunoreactivity were found to be decreased in the pancreatic islets of mice with type 1 diabetes, while increased in the islets of diet-induced obese mice with T2DM [10]. Human islet NUCB2 mRNA was reduced in T2DM subjects compared those in the controls [9]. NUCB2 protein expression level was significantly decreased in the hypothalamus of Tsumura Suzuki obese diabetes mice compared with the control mice [13]. In addition, plasma nesfatin-1 concentrations were significantly decreased in T2DM patients compared with healthy controls [5, 6]. However, another study demonstrated increased plasma nesfatin-1 in patients with newly diagnosed T2DM [14]. The explanation for these conflicting data is unclear but may be attributable to differences in disease advancement, populations, or assays applied.

Recently, the c.1012C>G polymorphism of NUCB2 gene was found to be correlated with obesity [7, 8]. Obesity is a risk factor of developing T2DM. Therefore, we hypothesized that the c.1012C>G polymorphism of NUCB2 gene may be also correlated with the risk of developing T2DM. The present study indicated that the c.1012C>G polymorphism of NUCB2 gene was associated with a decreased risk of developing T2DM. NUCB2 is considered to be a susceptibility gene for T2DM. The c.1012C>G polymorphism of NUCB2 could be utilized as a genetic marker to assess the risk of developing T2DM. However, when considering the blood pressure as the adjusting factor, the c.1012C>G polymorphism of NUCB2 was not correlated with the risk of developing T2DM. Traditionally, blood pressure is not a potential risk factor for T2DM. Therefore, we think blood pressure is confounding factor and should be excluded. The c.1012C>G polymorphism of NUCB2 gene was demonstrated to be associated with the risk of developing T2DM.

The potential limitations of these data merit consideration. First, as our study was conducted in a sample of Chinese Han population, extrapolation of the data to other ethnic groups should be done with great caution. Second, the study population was relatively small. Therefore, our results require further investigation in a larger sample size.

## Conclusions

In conclusion, this study revealed that the c.1012C>G polymorphism of NUCB2 gene was associated with the risk of developing T2DM. The c.1012C>G polymorphism of NUCB2 gene is suggested to be an independent genetic marker of the risk of T2DM.

## Abbreviations

T2DM: type 2 diabetes mellitus
NUCB2: nucleobindin 2
FPG: fasting plasma glucose
P_2h_PG: 2-h postprandial plasma glucose
SBP: systolic blood pressure
DBP: diastolic blood pressure
TG: triglycerides
TC: total cholesterol
HDL-C: high-density lipoprotein cholesterol
LDL-C: low-density lipoprotein cholesterol
BMI: body mass index
PCR: polymerase chain reaction
SD: standard deviation
